# *Pasteurella multocida* Involved in Respiratory Disease of Wild Chimpanzees

**DOI:** 10.1371/journal.pone.0024236

**Published:** 2011-09-08

**Authors:** Sophie Köndgen, Michaela Leider, Felix Lankester, Astrid Bethe, Antina Lübke-Becker, Fabian H. Leendertz, Christa Ewers

**Affiliations:** 1 Research Group Emerging Zoonoses, Robert Koch-Institut, Berlin, Germany; 2 Conservation and Science Department, Lincoln Park Zoo, Chicago, Illinois, United States of America; 3 Veterinary Faculty, Institute for Microbiology and Epizootics, Freie Universitaet Berlin, Berlin, Germany; 4 Department of Primatology, Max Planck Institute for Evolutionary Anthropology, Leipzig, Germany; Universidad Nacional, Heredia, Costa Rica

## Abstract

*Pasteurella multocida* can cause a variety of diseases in various species of mammals and birds throughout the world but nothing is known about its importance for wild great apes. In this study we isolated *P. multocida* from wild living, habituated chimpanzees from Taï National Park, Côte d'Ivoire. Isolates originated from two chimpanzees that died during a respiratory disease outbreak in 2004 as well as from one individual that developed chronic air-sacculitis following this outbreak. Four isolates were subjected to a full phenotypic and molecular characterisation. Two different clones were identified using pulsed field gel electrophoresis. Multi Locus Sequence Typing (MLST) enabled the identification of previous unknown alleles and two new sequence types, ST68 and ST69, were assigned. Phylogenetic analysis of the superoxide dismutase (*sodA*) gene and concatenated sequences from seven MLST-housekeeping genes showed close clustering within known *P. multocida* isolated from various hosts and geographic locations. Due to the clinical relevance of the strains described here, these results make an important contribution to our knowledge of pathogens involved in lethal disease outbreaks among endangered great apes.

## Introduction

*Pasteurella multocida* is a gram negative coccobacillus which colonizes the nasopharynx of many wild and domestic animals. It has a wide disease and host spectrum, ranging from clinical conditions where it is considered a primary pathogen, such as hemorrhagic septicaemia in cattle and fowl cholera in birds, through to conditions where it can behave as a secondary invader, for example in cases with pneumonic lesions [1], [2].

Research on *P. multocida* has to date primarily focussed on strains from livestock, poultry or companion animals. In humans, however, there are few data on *P. multocida* which is typically absent from the normal human bacterial flora. When human infections do occur they are predominantly as a result of bites or scratches by dogs and cats [3]–[6]. *P. multocida* may also cause upper respiratory tract infections, including sinusitis, epiglottitis and pharyngitis [3], [6]. In rare cases, lower respiratory tract infections, such as pneumonia and tracheobronchitis can develop, usually in individuals with underlying pulmonary disease [7]–[9]. *P. multocida* is also an infrequent cause of systemic infections such as meningitis and septicaemia, especially in the very young, the elderly and the immunocomprised [6], [10].

In nonhuman primates (NHP), respiratory tract infections due to *P. multocida* have been described for various species held in captivity [11]–[13]. For example, analysing respiratory tract disease among a dynamic laboratory colony including >8000 animals and 10 different species of NHPs, *P. multocida* was among the major bacterial pathogens isolated [11]. Together with *Streptococcus pneumoniae*, *P. multocida* was the most common bacterial isolate from the respiratory tract of nearly 2500 macaques (*Macaca fascicularis* and *M. mulatta*) that died from pneumonia [14], and in the South American owl monkey (*A. trivirgatus*) *P. multocida* was found as the principle respiratory pathogen responsible for disease [11]. *P. multocida* has also been associated with air-sacculitis, a clinical condition that can be characterised by the continuous accumulation of mucoserous or purulent fluid into the laryngeal air sacs which has been reported in several captive primate species, including great apes [15]–[18]. Apart from respiratory tract disease, *P. multocida* has been reported to be associated with septicaemia in Cebus monkeys (*Cebus albifrons*), septicaemia and meningitis in squirrel monkeys (*Sarmiri sciureus*) and several systemic suppurative diseases in owl monkeys (*Aotus trivergatus*) [19]–[22].

Whether pasteurellosis in NHPs arises from commensal bacteria or from bacteria transmitted from other animals has been barely investigated. There is only one study where *P. multocida* was part of the pharyngeal flora in healthy wild-born baboons [23]. So far, no data exist about pasteurellosis in wild NHPs.

The present study reports on the isolation of *P. multocida* from wild, human-habituated, chimpanzees (*Pan troglodytes verus*) living in the Taï National Park, Cote d'Ivoire. Isolates were obtained from victims of an outbreak of respiratory disease caused by human metapneumovirus (HMPV), *Sc. pneumoniae* and *P. multocida* [24], [25] and a chimpanzee showing air-sacculitis. Hence, in order to investigate *P. multocida* from wild living chimpanzees in more detail, the obtained isolates were subjected to a broad phenotypic and molecular characterisation.

## Results

Isolation of *P. multocida* was achieved for three different individuals (see table 1): three isolates originated from two females that died in the respiratory outbreak in 2004 (IMT18907, IMT18908, both from “Virunga”, and IMT18909, from “Ophelia”). Samples from the third victim of the 2004 outbreak were not cultivatable but had been identified as *P. multocida*-positive by PCR previously [24]. Another isolate was obtained from a chimpanzee called “Sagu” who was immobilised in 2009 (IMT18890). All isolates were confirmed as *P. multocida* by PCR amplification of the species specific gene fragment *kmt1* [26].

**Table 1:** Origins and characteristics of *Pasteurella multocida* isolated in this study.

P. multocida isolate	Individual	Clinic/ pathology	Sample material	Sampling date	PFGE pattern	ST	Further pathogens identified
IMT18907	Virunga	Pneumonia	Lung tissue	March 2004	1	68	Sc. pneumoniae^a^; HMPV^a^
IMT18908	Virunga	Pneumonia	Lung tissue	March 2004	2	69	Sc. pneumoniae^a^; HMPV^a^
IMT18909	Ophelia	Pneumonia	Lung tissue	March 2004	2	69	Sc. pneumoniae^a^; HMPV^a^
IMT18890	Sagu	Air-sacculitis	Pus	May 2009	1	68	Enterobacter sp.a, b; Prevotella sp.^b^

a Identified by PCR [24], [25].

b Identified by cultivation and subsequent phenotypical characterization.

HMPV  =  Human Metapneumovirus

### Phenotypic characterisation

#### Biochemistry

All isolates fulfilled the basic biochemical characteristics of *P. multocida*: they were positive for oxidase, catalase, indole and ornithine decarboxylase and showed no urease activity. Isolates were further characterised by fermentation reactions of different carbohydrates (table 2). The isolates IMT18907 and IMT18890 differed from the isolates IMT18908 and IMT18909 in the ability to ferment D-xylose. Fermentation reactions were also considered for the typing of the subspecies; here, variations in D-sorbitol, dulcitol and L-arabinose have been reported to be of taxonomic relevance [27]. However, due to conflicting fermentation patterns it was not possible to assign the subspecies: all isolates were dulcitol-negative and D-sorbitol-positive (characteristic for the subspecies *P. multocida* ssp. *multocida*) but positive for the fermentation of L-arabinose (characteristic for *P. multocida* ssp. *gallicida*).

**Table 2:** Biochemical profiles of *P. multocida* isolates from chimpanzees.

	IMT 18907+18890	IMT 18908+18909
**Oxidase**	+	+
**Catalase**	+	+
**Ornithine carboxylase**	+	+
**Indole**	+	+
**Urease**	−	−
**Trehalose**	+/−	+/−
**Maltose**	−	−
**Saccharose**	+	+
**D-Xylose**	−	+
**L-Arabinose**	+	+
**D-Mannitol**	+	+
**D-Sorbitol**	+	+
**Dulcitol**	−	−

+: positive; −: negative; +/−: ambiguous result.

#### Antimicrobial susceptibility testing

To predict the response to antimicrobial therapy and to get hints for anthropogenic influence, the isolates were tested against 16 antimicrobials by agar diffusion tests. The tested panel included β-lactam antimicrobials, aminoglycosides, fluoroquinolones, tetracyclines, sulfadimidine, lincosamide, polymyxine and chloramphenicole. Only minor differences between isolates were observed and resistance was generally low: all isolates were resistant to clindamycin and intermediate susceptible to tetracycline. Isolate IMT18890 was additionally resistant to sulfamethoxazole/trimethoprim (table 3).

**Table 3:** Antibiogram of *P. multocida* isolates (according to the standards given by CLSI [56]).

	IMT 18907	IMT 18890	IMT 18908	IMT 18909
**ß-lactamases**				
Amoxicillin-clavulanic acid	S	S	S	S
Ampicillin	S	S	S	S
Penicillin	S	S	S	S
Cefalexin	S	S	S	S
Cefazolin	S	S	S	S
Cefovecin	S	S	S	S
**Aminoglycosides**				
Amikacin	S	S	S	S
Gentamycin	S	S	S	S
**Fluoroquinolones**				
Enrofloxacin	S	S	S	S
Marbofloxacin	S	S	S	S
**Tetracyclines**				
Doxycycline	S	S	S	S
Tetracycline	I	I	I	I
**Sulfadimidine**				
Sulfamethoxazole/trimethoprim	S	R	S	S
**Lincosamide**				
Clindamycin	R	R	R	R
**Polymyxine**				
Polymyxin B	S	S	S	S
**Chloramphenicole**	S	S	S	S

S: susceptible; R: resistant; I: intermediate.

### Molecular characterisation

#### Macrorestriction analysis and pulsed-field gel electrophoresis

**Figure 1:**
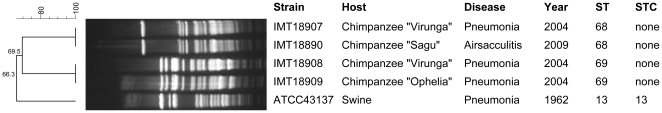
Dendrogram showing macrorestriction patterns of *P. multocida* isolates after digestion with *SmaI*. Cluster analysis of Dice similarity indices (UPGMA) was exerted to generate a dendrogram depicting the relationships among PFGE profiles using the BioNumerics software (optimisation 0.5%, position tolerance 1.0%). *P. multocida* reference strain ATCC 43137 served as methodological control.

To differentiate the *P. multocida* isolates macrorestriction and subsequent pulsed-field gel electrophoresis (PFGE) was performed: Using *SmaI*, two patterns were distinguished, differentiating between the isolates IMT18907 and IMT18890 (referred to as clone 1), and between IMT18908 and IMT18909 (referred to as clone 2) (figure 1). Restriction with *ApaI* worked only for isolates IMT18907 and IMT18890 and showed unique band patterns which confirmed their clonal nature (data not shown), whereas the isolates representing clone 2 could not be digested with that enzyme repeatedly.

#### Capsular type and virulence-associated gene profile

For further characterisation, the presence of three capsule biosynthesis genes and 13 virulence-associated genes (VAGs) was examined by PCR. All isolates were of capsular type A. The VAG pattern is shown in table 4 and differed between isolates IMT18907/IMT18890 and IMT18908/IMT18909, which is in agreement with pheno- and genotypic differences described above. Most of the regularly distributed VAGs could also be detected in the chimpanzee isolates: genes coding for outer membrane proteins (*ompH* and *oma87*), type 4 fimbriae (*ptfA*), superoxide dismutases (*sodA*, *sodC*), and iron acquisition related factors (*exbB/tonB*) were present in all tested isolates. Isolates were also positive for the *hgbB* gene which is coding for a hemoglobin binding protein and for *nanB* as a colonization-related gene. Differences were observed in the presence of *hgbA*, a further hemoglobin binding protein which was only detected in IMT18907/IMT18890 and the neuraminidase gene *nanH*, which was merely present in IMT18908/IMT18909. *PfhaB*, which encodes an adhesion-related factor called *Pasteurella* filamentous hemagglutinin, was only observed in isolates IMT18907/18890. None of the isolates harboured the genes coding for a dermonecrotoxin (*toxA*) and a transferrin binding protein (*tbpA*), respectively.

**Table 4:** Virulence-associated gene profiles of *P. multocida* isolates from chimpanzees.

Virulence-associated factor	Gene(s)	IMT 18907+18890	IMT 18908+18909
**Capsule**			
Capsular type A	*hyaD/C*	+	+
Capsular type D	*dcbF*	−	−
Capsular type F	*fcbD*	−	−
**Dermonecrotic toxin**	*toxA*	−	−
**Iron acquisition factors**			
Transferrin binding protein	*tbpA*	−	−
ExbB-ExbD-TonB-Locus	*exbB/tonB*	+	+
Hemoglobin binding proteins	*hgbA*	−	+
	*hgbB*	+	+
**Enzymes**			
Neuraminidase	*nanB*	+	+
	*nanH*	−	+
Superoxid dismutases	*sodC*	+	+
	*sodA*	+	+
**Outer membrane proteins**			
Oma87	*oma87*	+	+
OmpH	*ompH*	+	+
**Adhesion related genes**			
Filamentous hemagglutinin	*pfhA*	+	−
Type 4 fimbriae	*ptfA*	+	+

+: positive; −: negative.

#### Phylogenetic analysis of the sodA gene

**Figure 2:**
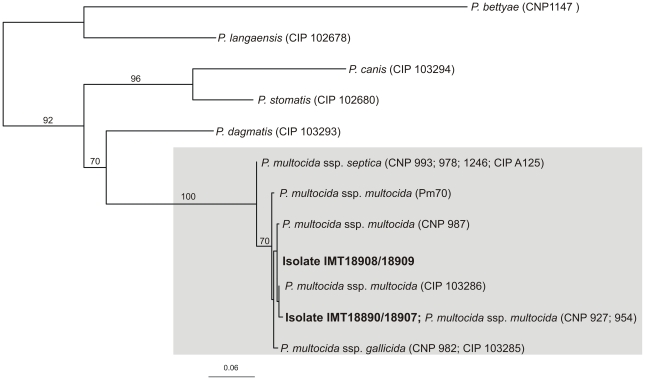
Phylogenetic analysis of the *sodA* gene. The tree was built using the maximum-likelihood method from an analysis of *sodA* sequences (452 bp) from the chimpanzee's isolates and sequences obtained from GenBank. Isolates belonging to the species *P. multocida* are boxed grey; the chimpanzees isolates are written in bold. Taxon labels indicate species and strain number, including strains with identical *sodA* sequences (species and subspecies assignments are according to Gautier et al. (2005) [28]). *P. langaensis* and *P. bettyae* were used as outgroup. Bootstrap values were calculated with 500 replicates and are given in percent.

To determine the subspecies, a phylogenetic tree for the *sodA* gene was constructed (figure 2). The topology of the *sodA* tree shows that different *P. multocida* reference strains are grouping together according to their subspecies delineation; however, statistical support was only given for the branching of *P. multocida* ssp. *septica* on the one hand and *P. multocida* ssp. *multocida* and ssp. *gallicida* on the other hand. The chimpanzee isolates group closest with strains of the subspecies *P. multocida* ssp. *multocida* and, in the case of IMT18907/IMT18890, *sodA* sequences were even identical with strains that had been classified as subspecies *multocida* (strain CNP 927 and CNP 954 [28]).

#### Multi Locus Sequence Typing (MLST)

**Figure 3:**
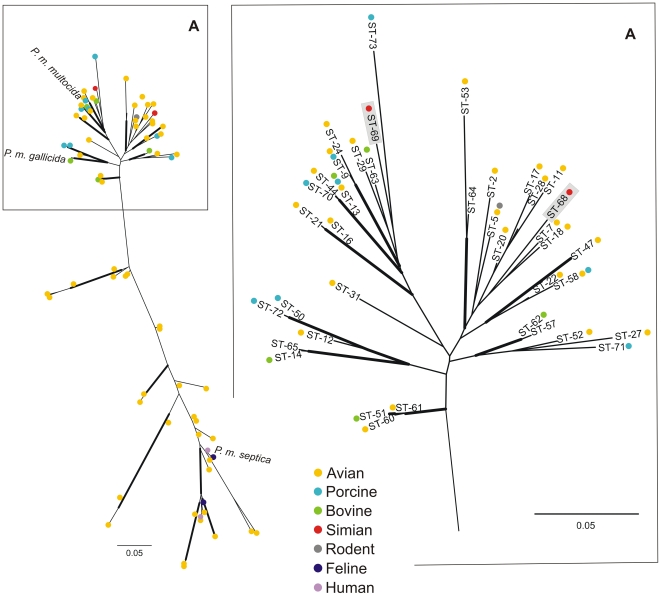
Radial Maximum Likelihood trees constructed with concatenated housekeeping gene allele sequences used for MLST analysis. Shown left is the complete MLST tree constructed with 73 sequence types (STs) and the position of the type strains for the *P. multocida* subspecies (*P. multocida* ssp. *septica*: CIPA125; *P. multocida* ssp. *multocida*: NCTC10322; *P. multocida* ssp. *gallicida*: NCTC10204). The position of the chimpanzee isolates (boxed grey) is displayed in the subtree (A). STs are labelled with coloured dots indicating the isolation sources and ST numbers. Bold branches indicate for bootstrap values >70%.

According to MLST analysis [29] we found four new allele types in est, gdh, mdh and pgi loci in both chimpanzee isolates belonging to clone 1 and also four new allele types in est, pgi, pmi and zwf loci in clone 2 (table 5). Only one allele, adk, was identical among all four isolates. Based on this allelic profile, two new sequence types (STs) were assigned: ST68 and ST69. To further analyse the genetic relationship between the chimpanzee isolates, concatenated gene sequences from both STs were compared to the available P. multocida STs from the MLST database (http://pubmlst.org/pmultocida/). Based on the assumption that there is no major impact of recombination among the housekeeping loci of the scheme used here [29], a maximum likelihood radial tree was constructed (figure 3) using the concatenated sequences of 73 STs which were colour-coded according to the host they had been isolated from. Based on the tree topology, two main groups can be distinguished: one group consists of isolates originating from birds, humans and cats and includes a type strain of the subspecies P. multocida ssp. septica (CIPA125). The other group (shown partly as a subtree in figure 3A) includes strains from various hosts (mainly birds, cattle, and pigs) and includes type strains of the subspecies P. multocida ssp. multocida (NCTC10322) and P. multocida ssp. gallicida (NCTC10204). The STs found in the chimpanzees group together with strains of the latter group.

**Table 5:** Allelic profile of *P. multocida* isolates according to MLST analyses (as defined by the scheme published by Subaaharan et al. (2010) [29]).

Isolate	Allel number for gene fragment*							ST
	*adk*	*est*	*gdh*	*mdh*	*pgi*	*pmi*	*zwf*	
IMT18907	21	**33**	**20**	**17**	**42**	26	4	68
IMT18890	21	**33**	**20**	**17**	**42**	26	4	68
IMT18908	21	**40**	11	14	**41**	**34**	**31**	69
IMT18909	21	**40**	11	14	**41**	**34**	**31**	69

*Allele numbers written in bold assign for new alleles.

## Discussion

This is the first description of *P. multocida* isolated from wild great apes. Three isolates found in the lungs of the deceased chimpanzees were compared to an isolate found in the pus from a chimpanzee that underwent surgery due to air-sacculitis. Phenotypically, all isolates matched the properties of previously described strains of the species *P. multocida*. PCR analysis showed that all isolates were of capsular type A. The capsular type in *P. multocida* is assumed to play a likely role in host, and to a greater extent, in disease specificity. Capsular type A strains are predominantly associated with fowl cholera and upper and lower respiratory tract diseases in ruminants, pigs and rabbits [30]–[32]. There is scarce information on the capsule type of *P. multocida* isolated from non-human primates but for humans it has been shown that their distribution differs according to the site of infection and that capsular type A strains are often associated with respiratory tract infections [33], [34]. This corresponds well with the clinical picture we observed in the deceased chimpanzees.

*P. multocida* constitutes a heterogeneous species and virulence features are divergent. However, efforts have been made to correlate several virulence gene patterns to distinct hosts (i.e. cattle and swine) and specific diseases [31], [35]. The chimpanzee isolates showed virulence gene patterns that have already been observed among isolates linked with respiratory diseases in other hosts. While the absence of dermonecrotoxin gene *toxA* (as an essential factor in the pathogenesis of atrophic rhinitis in swine) and of the transferrin binding protein gene *tbpA* (which so far has been almost exclusively associated with ruminant *P. multocida* isolates) was not unexpected, the presence of the remaining VAGs in the wild chimpanzee isolates is comparable to that observed among respiratory tract isolates from domestic animals. Consistent with that, the *Pasteurella* filamentous hemagglutinin gene *pfhaB*, which was found to be irregularly distributed among animal isolates in previous studies [31], [35], was only detected in one of the chimpanzee clones. Initial epidemiological data on porcine strains [35] hint towards an association of this putative adhesin with the virulence capacity of the strains.

To determine the subspecies of the isolates, we took into account the results from the phylogenetic analysis of the *sodA* gene as well as from the fermentation reactions. However, none of these methods allowed an unambiguous identification of the chimpanzee's strains: the *sodA* sequences grouped firmly within strains belonging to both the subspecies *multocida* and *gallicida*, and the conflicting results of the biochemical characterization, including dulcitol-negative, sorbitol-positive (as seen in ssp. *multocida* [27]), and arabinose-positive reactions (as seen in ssp. *gallicida* [27], [36], [37]) did not allow for the identification of the subspecies either. However, conflicting results from both molecular and phenotypic data have been described before [38]–[40], which shows that the precise typing of the subspecies is complex and has not been fully clarified yet.

MLST is a highly specific, sensitive and stable tool and is currently one of the ”gold standards” used for typing of bacteria. The MLST scheme used here has only recently been developed [29] and at the time of writing consisted of 177 isolates representing 73 STs which built six clonal complexes (with a clonal complex being defined as STs that shared 6 or more loci). Our analysis revealed that each clone represented four new alleles and based on the allelic profiles two new STs were assigned (ST68 and ST69), which did not belong to any ST complex. To further analyse the genetic relationships, concatenated gene sequences from the chimpanzees STs were compared to all further STs from the MLST database (http://pubmlst.org/pmultocida/). Both STs group within strains originating from various hosts, related more closely to the reference strains of the subspecies *P. multocida* ssp. *multocida* and ssp. *gallicida*. However, it should be noted that this database is still a work in progress. So far it consists mostly of isolates obtained from domesticated animals (mainly of avian origin) and from a limited number of geographical locations. Based on the present data, it can be stated that although the chimpanzees strains show phylogenetic differences they are still embedded in the global epidemiology of *P. multocida* but further conclusions about host- or geographic associations must not be drawn until additional data from African countries as well as from primates have been included in the MLST database.

The results from the antibiotic susceptibility testing can be basically rated as positive, since isolates were only resistant to clindamycin. This resistance is observed frequently [41] and is most likely indigenous in *P. multocida*. Interestingly, isolate IMT18890 (clone 1), which was isolated from the air sacs of the immobilised chimpanzee in 2009, showed additional resistance to sulfamethoxazole/trimetho prim, a drug never used in chimpanzees of the study groups. Since the corresponding isolate from 2004 (IMT18907; clone 1) was susceptible to that agent, the resistance has been probably acquired by external sources. The origin of this resistance is difficult to determine. However, since the sulphonamide- and the trimethoprim resistance gene can be encoded on a plasmid [42], [43], it is possible that it has spread into the forest through various interspecies transmission events. For *E. coli*, such a spread of resistance genes has already been described in chimpanzees and gorillas in Uganda [44], [45] and with regard to the previously suggested transmission of *P. multocida* between cattle, pigs, and poultry (figure 3;[46]), the concurrent dissemination of resistance determinants might appear in wildlife as well. In most animals, infections with *P. multocida* are primarily transmitted by the respiratory route via direct contact to infectious secretions, inhalation of aerosols or by uptake of contaminated water or food [47], [48]. The Taï chimpanzees share their habitat and food sources, such as fruit trees, with a number of different mammals and bird species, including ruminants, rodents and also carnivores, most of them known as carriers of *P. multocida*, which may represent a source of infection for the chimpanzees.

Pasteurellosis in captive NHPs often occurs when local and systemic defense mechanisms are impaired. Predisposing factors include stress induced by transportation, crowding or by the damaging effects of respiratory viral infections [49]. In addition, NHPs have developed *P. multocida* infections secondary to surgical procedures or chronic catheterization [23]. The respiratory outbreak among the chimpanzees studied here was caused by a multifactorial infection, where HMPV as well as *Sc. pneumoniae* and *P. multocida* were found in the lung tissue. It is difficult, therefore, to determine the actual role of *P. multocida* in the causation of disease. However, it is possible that the underlying viral infection paved the way for the secondary bacterial infection, which ultimately could have caused the lethal outcome.

The *P. multocida* isolate recovered from the purulent discharge of the air sac of a male animal of the same community in 2009 matched with one of the strains involved in the respiratory outbreak of 2004 (IMT18907/18890; clone 1; ST68). This, in combination with the date of the onset of clinical symptoms (end of 2004), suggests that the chronic air-sacculitis had resulted from the former respiratory disease. Similar data have been shown in studies on air-sacculitis in orang-utans and chimpanzees respectively, where several affected animals had a history of recent acute upper respiratory tract infection [15], [18]. Apart from *P. multocida* we also isolated *Enterobacter* sp. and *Prevotella* sp. from the air sacs, suggesting that *P. multocida* was not the single cause. This is in agreement with the literature, where air-sacculitis has been associated with mixed infections of enteric organisms [50].

The strains of *P. multocida* isolated from the Taï chimpanzees reported here were involved in acute and/or chronic respiratory disease. Since all the reported cases were caused by a mix of viral and/or bacterial pathogens, it seems probable that *P. multocida* might have acted as secondary pathogen. However, the question if the chimpanzees are frequent carriers of *P. multocida*, or if they are exposed to a permanent transmission risk by external sources cannot be answered here, since an evaluation of the carrier state would require a systematic and invasive sample collection (including swab samples) from healthy chimpanzees, which is not feasible without disturbing their natural behaviour.

To summarize, this is the first description of *P. multocida* involved in diseases of wild living chimpanzees. To date, there is only few detailed molecular data on strains from Africa and no data on wild NHP-associated strains, hence it was interesting that the strains analysed here were found to be similar to known strains of *P. multocida.* Both clones were found in the lungs of chimpanzees that died in the course of a respiratory tract disease, caused by a mix of viral and/or bacterial pathogens. Among the Taï chimpanzees, *P. multocida* was not detected in any further case of respiratory infection before and after the cases described here [24], [25]. However, acute respiratory disease is considered one of the major threats for endangered great apes [25], [51], [52], therefore a profound characterisation of the pathogens involved is of great relevance for conservation strategies. For example, data on antimicrobial susceptibility may help to provide adequate treatment and thus mitigate the impact of future disease outbreaks.

## Materials and Methods

### Ethics statement

This research was conducted entirely on free-ranging chimpanzees and under permission of the wildlife authorities and research ministries of Côte d'Ivoire. Samples were collected from chimpanzees that died of natural reason [25] and in one case obtained from an individual on which surgery had to be performed due to a live threatening infection. No animal was anaesthetised or touched for the sole purpose of sample collection.

### Sources of isolates

Samples were collected from wild chimpanzees from Taï National Park, Côte d'Ivoire that are known individually as a result of a project focusing on wild chimpanzee behaviour [53]. The chimpanzees are followed on a daily basis by researchers. Whenever a chimpanzee shows signs of weakness or disease, the individual is followed continuously and detailed data on the type and intensity of clinical symptoms are recorded [54].

In March 2004, one community consisting of 44 chimpanzees was hit by a respiratory disease outbreak. Morbidity and mortality were high during this outbreak: all 28 chimpanzees observed in the course of this outbreak were seen with symptoms of respiratory illness and out of these eight died. Three of the eight fatalities (“Ophelia”, “Virunga” and “Orest”) were found shortly after death and consequently necropsies were able to be performed and tissue samples, collected therein, were stored in liquid nitrogen [25], [54]. In addition, in May 2009, a male member of the same chimpanzee community (“Sagu”) was immobilised for the purpose of surgically treating a case of air-sacculitis which had been developing since 2004 (unpublished data). Swabs were taken from the purulent discharge of the air sac and transported in charcoal Amies Medium at +8°C (BD, Heidelberg, Germany). Samples were exported to Germany under permission of CITES authorities and according to national regulations.

### Phenotypic characterisation

#### Culture conditions and biochemical analyses

Lung tissue was placed in brain heart infusion (BHI) and incubated overnight at 37°C before inoculation on tryptic soy yeast extract (TSYE) agar supplemented with 5% of defibrinated sheep blood (Oxoid, Wesel, Germany). Swab samples taken from the air-sacculitis were inoculated on the same media directly. Isolates were identified as P. multocida using standard biochemical procedures, including production of catalase, oxidase, and indole, urease activity, as well as production of ornithine decarboxylase [55]. All isolates were further characterised by fermentation reactions to eight carbohydrate substrates: trehalose, maltose, saccharose, D-xylose, L-arabinose, D-mannitol, D-sorbitol and dulcitol [55]. In case of ambiguous results, tests were performed repeatedly.

#### Antimicrobial susceptibility testing

Antimicrobial susceptibility was tested by agar diffusion test according to the standards given by the Clinical and Laboratory Standards Institute [56]. The antimicrobial agents tested included amoxicillin-clavulanic acid (20/10 µg), amikacin (30 µg), ampicillin (10 µg), cefalexin (30 µg), cefazolin (30 µg), cefovecin (30 µg), chloramphenicol (30 µg), clindamycin (2 µg), doxycyclin (30 µg), enrofloxacin (5 µg), gentamycin (10 µg), marbofloxacin (5 µg), penicillin (10 IU), polymyxin B (300 IU), sulfamethoxazole/trimethoprim (23,75/1,25 µg), and tetracycline (30 µg) (Becton Dickinson, Heidelberg, Germany). Escherichia coli ATCC 25922 served as the reference strain.

### Molecular characterisation

#### Macrorestriction analysis and PFGE

Isolates of P. multocida were grown overnight in BHI at 37°C and adjusted with PBS to an optical density (OD)600 of approximately 0.7. P. multocida ATCC 43137 (capsular type A) served as methodological control strain. One-and-a-half millilitres of culture was used for DNA preparation and centrifuged to obtain a bacterial pellet (8000 rpm for 5 min). The supernatant was discarded and the pellet re-suspended in 250 µl of PBS by vortexing. The bacterial suspension was warmed to 37°C. The 1.2% PFGE agarose (Peqlab Biotechnologie GmbH, Erlangen, Germany) was melted and equilibrated to 60°C. Two-hundred and fifty microlitres of agarose were added to the bacterial suspension and mixed thoroughly. The mixture was transferred to plug moulds (Bio-Rad Laboratories, Munich, Germany) and the agarose was allowed to solidify at 4°C for 20 min. Solidified plugs were incubated in 0.5 ml of EDTA-sarcosine buffer containing 0.45 mg proteinase K (Carl Roth GmbH & Co. KG, Karlsruhe, Germany) at 56°C overnight. Plugs were washed four times in 13 ml TE buffer for 30 minutes each at 4°C with gentle agitation. The plugs were halved and stored in 1 ml of fresh TE buffer. Prior to restriction, each plug was incubated with 0.2 ml 1 x restriction buffer for 30°C at room temperature for equilibration. One half of the plug was digested with ApaI restriction enzyme, the other with SmaI restriction enzyme (Fermentas GmbH, St. Leon-Rot, Germany). For ApaI, plugs were incubated overnight in 150 µl of 1 x restriction buffer containing 20 units of the restriction enzyme at 37°C. For SmaI, plugs were incubated overnight in 200 µl of 1 x restriction buffer containing 10 units of the restriction enzyme at 30°C. After digestion, the buffer was removed and plugs were then equilibrated in 0.5 ml TE buffer. The fragments were separated in a 1.2% agarose gel (Peq Lab, Erlangen; Germany) in 0.5 x TBE buffer by using a CHEF-DR III system (Bio-Rad Laboratories, Munich, Germany). The electrophoresis conditions for ApaI were 6 V/cm at 14°C for 22 h, ramping times were 1–30 s. Separation of SmaI fragments was conducted at 5.6 V/cm and pulse time was ramped from 2–5 s for 11 h and 20–40 s for 13 h. PFGE profiles were compared digitally using BioNumerics software (Version 4.6, Applied Maths, Belgium). Cluster analysis of Dice similarity indices based on the unweighted pair group method with arithmetic mean (UPGMA) was exerted to generate a dendrogram depicting the relationships among PFGE profiles.

#### DNA extraction

Bacterial DNA was extracted by a boiling procedure. Colony material was suspended in 50 µl of double distilled water, boiled for 10 minutes and centrifuged. Two microliters of the supernatant served as a template for PCR reactions. For the sodA PCR as well as for MLST analysis - where amplicons needed to be sequenced - total DNA was extracted using the Master PureTM Genomic DNA Purification Kit (Biozym Diagnostik GmbH, Hessisch Oldendorf, Germany) according to the manufacturer's recommendations.

#### PCR analysis, purification of amplicons and sequencing

All isolates were tested for the *Pasteurella multocida* species specific genomic sequence *kmt*1 [26]. PCR assays targeting the capsular biosynthesis genes and virulence associated genes (VAG) were performed using oligonucleotide primers, amplification conditions and reference strains published previously [31], [35], [57].

MLST analysis was performed according to a previously published scheme [29] using standard primers and protocols given on the mlst.net site (http://pubmlst.org/pmultocida_rirdc/info/primers.shtml). PCR fragments of the seven housekeeping genes *adk*, *pgi*, *mdh*, *gdh*, *est*, *pmi*, and *zwf* were obtained for all isolates and sequenced subsequently. New allele numbers and sequence types (STs) were assigned through the curator and entered in the MLST database (http://pubmlst.org/pmultocida/).

For phylogenetic and/or MLST analysis PCR products were purified using ExoSAP (USB Europe GmBH, Staufen, Germany). Sequencing was performed using the ABI Big Dye Termination Kit (Applied Biosystems, Weiterstadt, Germany) in a 377 DNA automated sequencer (Applied Biosystems), with all PCR products being sequenced on both strands. The *sodA* sequences were deposited in GenBank under accession numbers HQ003894- HQ003897.

#### Phylogenetic analysis

The phylogenetic tree for sodA was calculated with sequences collected from P. multocida strains available at the public GenBank database (http://www.ncbi.nlm.nih.gov/) and included all available sequences from strains that had been previously typed to the subspecies level [28]. Alignments were constructed using the ClustalW software program in BioEdit software version 7.0.9 [58] and sequences were collapsed into unique haplotypes using FaBox [59]. The final data set contained 12 taxa and 464 positions. Furthermore, phylogenetic analysis was performed using concatenated MLST allele sequences of all available STs from the MLST database (72 STs; 3696 positions (http://pubmlst.org/pmultocida_rirdc/)).

To determine the appropriate nucleotide substitution model, alignments were then exported into jModeltest v0.1.1 [60], [61]. According to the Akaike information criterion (AIC), comparisons of model likelihoods were most favourable to GTR+G (*sodA*), and GTR+I+G (MLST). Phylogenetic trees were built using the PhyML webserver (http://www.atgc-montpellier.fr/phyml/; [60]–[62]). Equilibrium frequencies, topology and branch lengths were optimised, the starting tree was determined using BioNJ and both nearest neighbour interchange (NNI) and subtree pruning and regrafting (SPR) algorithms of tree search were used (keeping the best outcome). Branch robustness was assessed by performing non-parametric bootstrapping with 500 replicates (*sodA*) and 100 replicates (MLST), respectively.
